# SAPM: Self-Adaptive Parallel Manipulator with Pose and Force Adjustment for Robotic Ultrasonography

**DOI:** 10.1109/TIE.2022.3220864

**Published:** 2023-10

**Authors:** Xianqiang Bao, Shuangyi Wang, Lingling Zheng, Richard James Housden, Joseph Hajnal, Kawal Rhode

**Affiliations:** School of Biomedical Engineering & Imaging Sciences, King’s College London, SE1 7EH, United Kingdom; State Key Laboratory of Management and Control for Complex Systems, Institute of Automation, Chinese Academy of Sciences, Beijing, 100190, China; Faculty of Engineering and Design, Kagawa University, Takamatsu 761-0396, Japan; School of Biomedical Engineering & Imaging Sciences, King’s College London, SE1 7EH, United Kingdom

**Keywords:** Robotic ultrasonography, automatic pose adjustment, constant forces/torques, mechanical measurement, operation safety

## Abstract

Robotic ultrasonography potentially acts as an essential aid to medical diagnosis. To overcome the limitations in robotic ultrasonography, in this paper, we proposed a novel self-adaptive parallel manipulator (SAPM) that can automatically adjust the ultrasound (US) probe pose to adapt to various contours of scanned areas, provide approximate constant operating forces/torques, achieve mechanical measurement, and cushion undesired produced forces. A novel parallel adjustment mechanism is proposed to attain automatic pose adjustment with 3 degrees of freedom (DOFs). This mechanism enables the US probe to adapt to different scanned areas and to perform the scanning with approximate constant forces and torques. Besides, we present a mechanical measurement and safety protection method that can be integrated into the SAPM and used as operation status monitoring and early warning during scanning procedures by capturing operating forces and torques. Experiments were carried out to calibrate the measurement and buffer units and evaluate the performance of the SAPM. Experimental results show the ability of the SAPM to provide 3-DoFs motion and operating force/torque measurement and automatically adjust the US probe pose to capture US images of equally good quality compared to a manual sonographer scan. Moreover, it has characteristics similar to soft robots that could significantly improve operation safety, and could be extended to some other engineering or medical applications.

## I Introduction

**Fig. 1:**
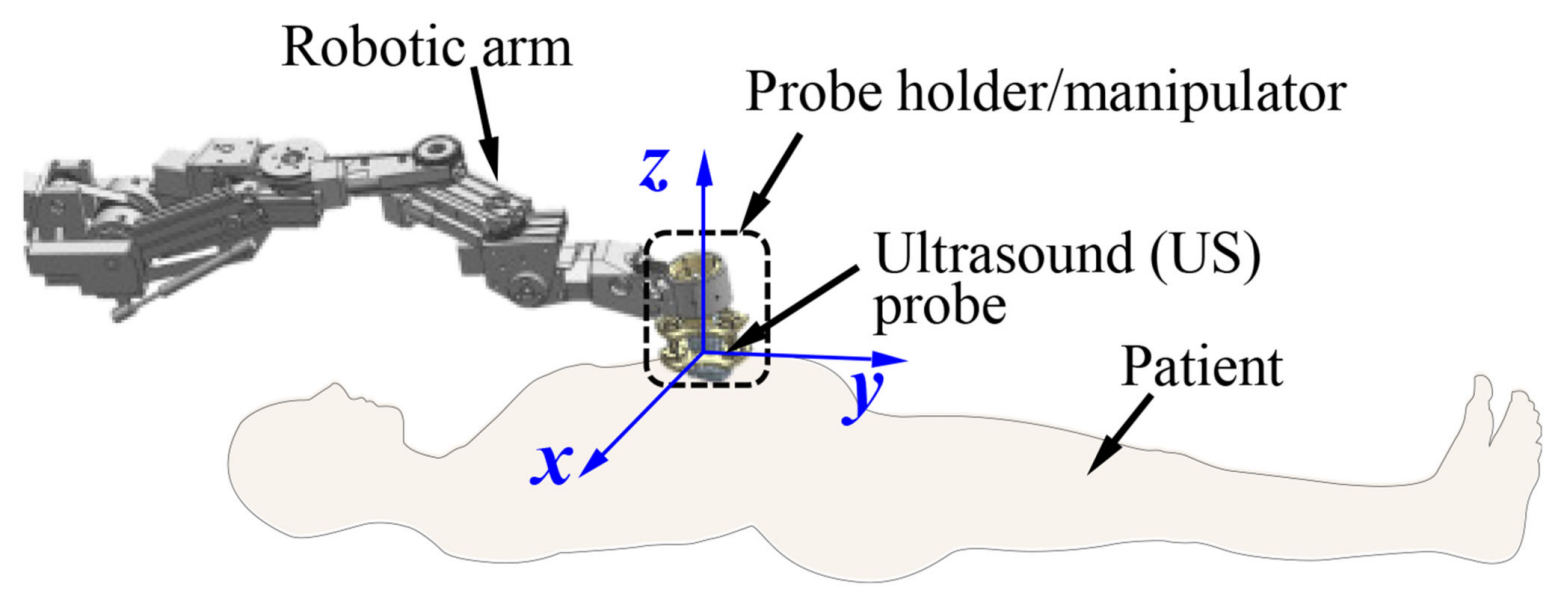
Diagram of the robotic ultrasonography.

Robotic ultrasonography has become a potentially effective tool in medical applications due to its precision, dexterity, and repeatability [1]–[2]. A commercial ultrasound (US) probe is commonly mounted on a robotic arm operated remotely by sonographers in robotic ultrasonography (Fig.1). During the scanning procedure, sonographers manipulate the robot system through the following three types of operations: (1) control the robotic arm and position its end near the target; (2) adjust the pose of the US probe to achieve satisfactory imaging angles; (3) control the contact forces and torques between the scanned areas and the US probe within a safe range (especially for the fetal imaging). The sonographers mainly concentrate on the second and third ones since they are critical operations for image acquisition and operation safety. In addition, the second and third operations are also concerned about the scanning difficulty, efficiency, and workload. With regard to operation (2), the pose of the US probe needs to be adjusted with the changes in abdominal contour, and the sonographers continuously focus on pose adjustment during the scanning. In addition, sonographers are required to maintain a specific pose for a long time for prolonged surgeries or scannings and would suffer from muscle fatigue [3]–[4]. For operation (3), the contact forces and torques increase immediately with the movement of the US probe toward the abdomen. It is difficult for sonographers to accurately determine the contact forces/torques and provide consistent contact forces/torques during the scanning [5]. Excessive contact forces/torques could lead to discomfort for patients and cause potential injuries, while inadequate contact forces/torques may result in low-quality images [6]–[7]. Therefore, operations (2) and (3) introduce operation safety and high-quality image acquisition issues, increase sonographers’ workload, and decrease scanning efficiency.

Various researchers have been devoted to studying pose/position adjustment, force/torque control, and operation safety in robotic ultrasonography. A. Vilchis *et al*. developed a cable-driven robot that can carry US probes for remote echographic examination [8]. The US probe positions are adjusted to contact the scanned areas by cable movement. The robot belongs to a slave manipulator of a master-slave system controlled by operators but cannot adapt its pose automatically. Z. Jiang *et al*. proposed an optimization algorithm to align the US probe to the normal surface at the point of contact [9]. This algorithm can automatically adjust the US probe pose to obtain good-quality orthopaedics US images. However, these reported studies mainly focus on the movement of the US probe but failed to control the contact forces or torques. For the contact force or torque control, in [10], a novel mobile tele-echography system was proposed to manipulate the US probe remotely according to the operation signals acquired from operators on the master side. The contact force can be controlled based on expert experience and a maximum allowable threshold. R. Tsumura *et al*. developed a robot that can operate the US probe and keep the contact force in a particular range using a constant spring and linear actuator [11]. Meanwhile, we also developed a customized spring-loaded ball clutch joint that can ensure operation safety by limiting the operating force in a certain range [12]. These proposed robots concentrated on limiting the contact forces rather than both forces and torques in a certain range, and additionally, did not integrate with the automatic pose adjustment of the US probe.

Many vision-based controllers were proposed to control the robot system employing various visual features, such as points [13], moments [14], and wavelets [15]. In [16]–[17], the authors proposed a novel US feedback-based method to control three DOFs of the robot, enabling the US probe to move around a complex shape like the breast. In [18], a vision-based approach allowing autonomous robotic US limb scanning was developed and experiments demonstrated its ability to capture the planned vascular structure on volunteers’ limbs. In our previous research, we proposed a rule-based end-point calculation method to position each individual joint of the US robot by abdominal surface mapping based on the abdominal contour captured by Kinect cameras [19]. However, these vision-based methods highly depend on the quality of the acquired images and the matching of positions. Even though various methods have been proposed to obtain high-quality images of scanned tissue or external contour, such as [20]–[21], safety issues will also be introduced without controlling the operating forces or torques.

In [22], Q. Huang *et al*. tried to employ a depth camera to capture the point cloud of the skin surface and two force sensors were used to measure the contact force between the emission plane and the scanned tissue for operation safety. J. Zhang *et al*. proposed a self-adaptive ultrasound scanning system to image the human spine [5]. This system can automatically perform the scanning and maintain the resultant force from 14 to 16 N on different spine segments. These researchers tried to integrate the force control and automatic pose adjustment into a system, but some issues remain. These robots adjusted the pose only in the vertical direction (*z*-direction in Fig. 1) and failed to maintain both force and torque. Besides, the pose adjustment and the force control were achieved by precise control of the robotic arm with sensors and actuators. These methods do not separate the contact force control and pose adjustment from the robotic arm control, which is not conducive to the control and function expansion of the robotic arm. The individual control method can enable the robotic arm to be controlled more straightforwardly with higher response accuracy since the robotic arm only needs to focus on position adjustment.

In our previous research, a novel soft robotic end-effector composed of soft fluidic actuators was proposed [7]. This end-effector can realize the 3D-pose adjustment but is unable to control the contact force or torques. In [23], we proposed a constant-force end-effector, and it can precisely generate constant operating forces and enable the US probe to adapt to the abdominal contours autonomously. However, the pose can be adjusted only in the vertical direction (*z*-direction) and only contact force is maintained. Generally, a manipulator with a simple structure and easy control, achieving both force and torque control, and automatic pose adjustment with multiple degrees of freedom (DOFs), would be a solution to the issues introduced by operations (2) and (3).

In this research, to overcome the limitations mentioned above and expand our previous study, we propose a self-adaptive parallel manipulator (SAPM) that can be mounted on the end of a robotic arm (e.g. the customized US robots we developed for the iFind project [19], [24], or any appropriate commercially-available robotic arm [25]) and has individual control relative to the robotic arm. The main contributions of this research are as follows.

(1)We propose a novel parallel adjustment mechanism to achieve automatic pose adjustment with 3 DOFs. This mechanism enables the US probe to adapt to different scanned areas and to perform the scanning with approximate constant forces and torques. It will be unnecessary for sonographers to care about the contours of scanned areas and values of the forces and torques, and therefore, sonographers’ workload could be decreased, and operation efficiency could be improved. Besides, operations with approximate constant operating forces and torques are beneficial to improve operation stability, facilitate the acquisition of high-quality US images, and improve the comfort for patients.

(2)A method for mechanical measurement and safety protection is proposed. This method is able to measure operating forces and torques that can be used as operation status monitoring and early warning during scanning procedures. Besides, this solution can cushion the undesired produced forces like soft robots, and thus, using the proposed SAPM instead of rigid end-effectors could significantly improve operation safety.

(3)We integrate various functions (i.e., automatic pose adjustment, mechanical measurement, constant operating forces/torques, and buffering) into a solution that requires simple structures and has low cost and good expandability. These features allow this proposed solution to be conducive to clinical application, and all design parameters can be simply modified to achieve applications in other fields. In addition, this solution is independent of the robotic arm and performs autonomous control, which is conducive to expanding other functions of the robotic arm.

The remainder of this paper is organized as follows. Section II describes the clinical requirements and details of the design, control, force analysis, and kinematics modelling of the proposed SAPM. Performance evaluation experiments are conducted, and corresponding results and discussions are presented in Section III. Finally, Section IV concludes this research.

## II Method

### A Clinical Requirements

In US scanning, US probes need to be applied with forces and torques to obtain satisfactory poses for image acquisition. The poses of US probes change with the contours of scanned areas. To investigate the required mechanical and motion data during the scanning, clinical studies were carried out at St Thomas’ Hospital, London, UK. (Study title: Intelligent Fetal Imaging and Diagnosis (iFIND)-2: Further US and MR Imaging, Study reference: 14/LO/1806). In the experiments, a standard US probe was used to perform the scanning and force/torque and position measurement devices were employed to capture the mechanical and motion data. A six-axis force sensor (Nano 17, ATI, USA) and an electromagnetic tracking sensor (Aurora, NDI, CA) were integrated with a bespoke probe holder (described in [26]) that was used to hold a US probe (X6-1, Philips, NL). The US probe was connected to a US scanner (EPIQ7, Philips, NL) for image acquisition. Trained sonographers performed fetal US scanning on pregnant women between 18 to 24 weeks of gestation. The operating forces and torques during scanning were recorded by the six-axis force sensor and the positions and orientations of the probe were captured by the electromagnetic tracking sensor. The data of six women were analyzed by extracting time ranges during which standard fetal anomaly views were imaged. Based on the results from the clinical data, to obtain no significant deterioration of the imaging view, a manipulator operating the US probe at least needs to 1) withstand an axial force of 8.01 N (in the *z*-direction) and torques of 150 mN·m around the *x*- and *y*-directions, and 2) provide axial displacement of 5.22mm (in the *z*-direction) and rotations of 5.08° around the *x*- and *y*-directions [7].

### B Design

#### 1 Overview

**Fig. 2:**
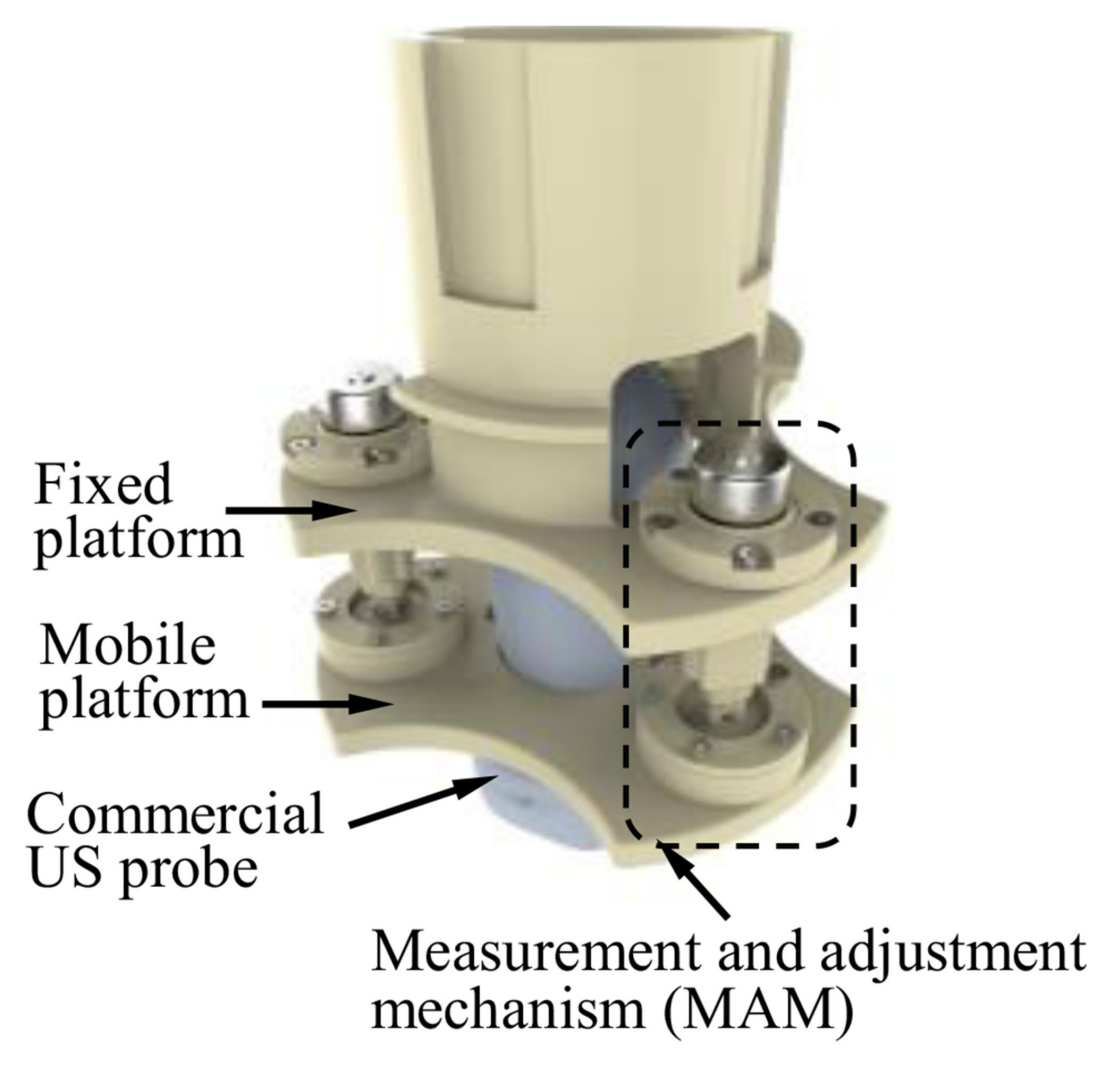
Diagram of the proposed self-adaptive parallel manipulator (SAPM).

The diagram of the developed SAPM is shown in Fig. 2. The SAPM consists of a fixed platform, a mobile platform, and three measurement and adjustment mechanisms (MAMs). The fixed platform is mounted on the end of a robotic arm that can be teleoperated by sonographers (shown in Fig. 1). It provides support to all the components of the SAPM. The MAM is capable of measuring forces and adjusting displacements. These three MAMs are evenly arranged in a ring, and parallel link the fixed platform with the mobile platform. The mobile platform grasps the US probe and its pose can be altered with the movement of the MAMs.

**Fig. 3:**
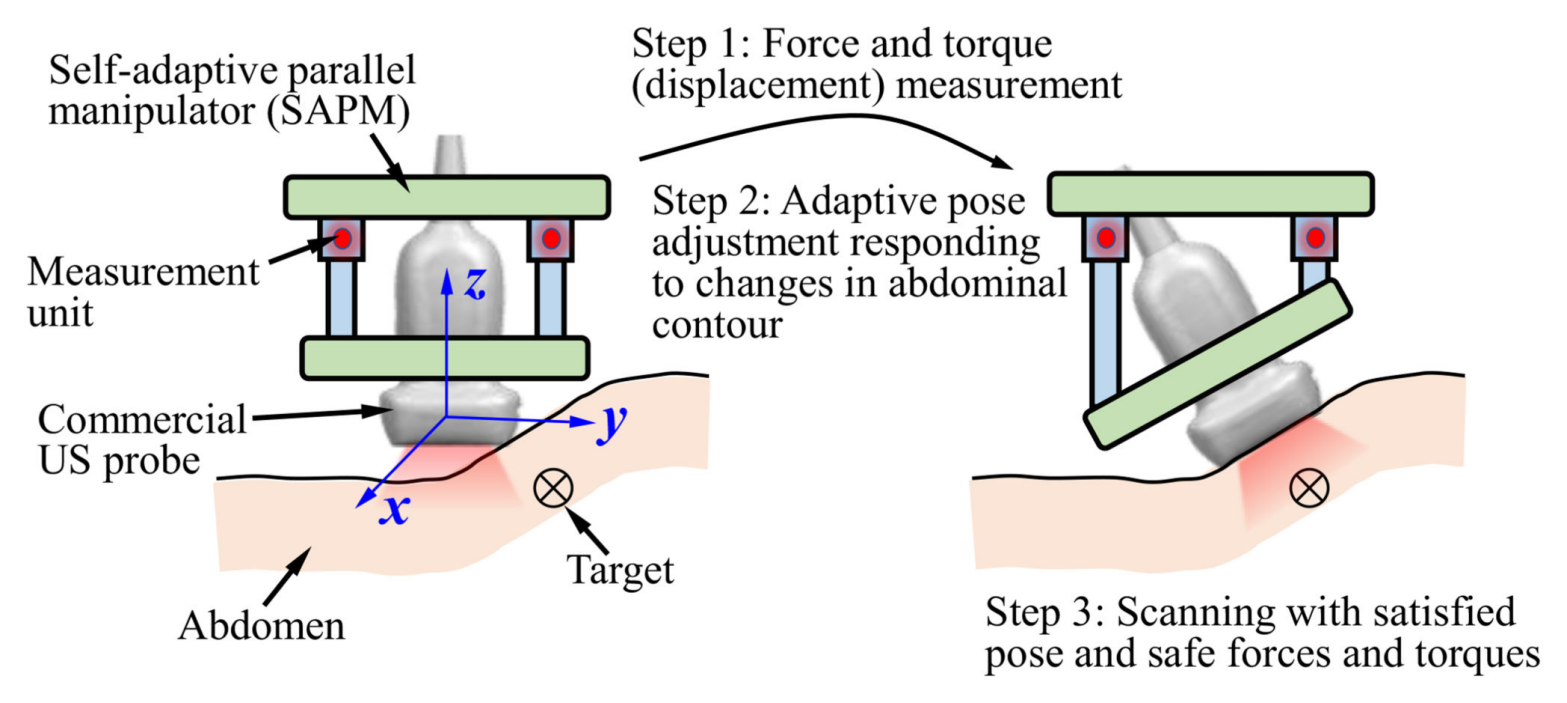
Adaptive adjustment workflow of the proposed SAPM.

The SAPM can 1) measure forces and torques, 2) keep the forces and torques constant (within a safe range), and 3) automatically adjust the pose according to the changes in abdominal contour. As shown in Fig. 3, the abdominal contour varies and the pose of the US probe will not be suitable for image acquisition. This situation is detected by the measurement units. Then, the MAMs start to run and change the pose of the mobile platform. With the measurement and feedback, the US probe achieves a satisfactory pose for imaging, and the contact forces and torques are also maintained within a safe range. When this proposed SAPM is used in the scanning, sonographers are able to concentrate all efforts on the observation of US images and target positioning (i.e., operation (1) discussed in Introduction), and do not need to pay attention to the operating forces and torques, abdominal contours, and the US probe pose (i.e., operations (2) and (3) discussed in Introduction), which will significantly improve the operation safety and efficiency and decrease the workload of sonographers.

#### 2 Structure

**Fig. 4:**
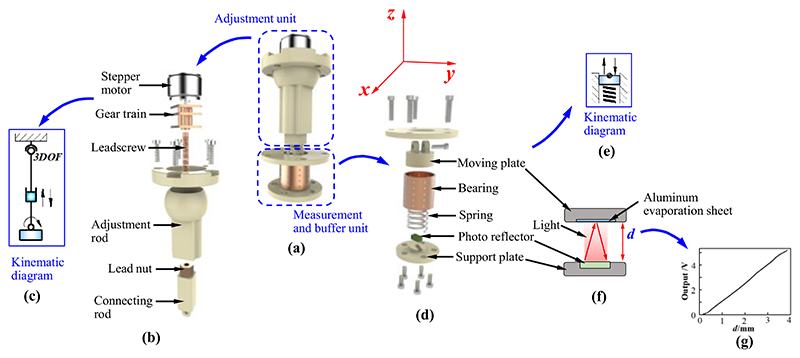
Design details of the measurement and adjustment mechanism (MAM): (a) overview; (b) exploded view of the adjustment unit; (c) kinematic diagram of the adjustment unit; (d) exploded view of the measurement and buffer unit; (e) kinematic diagram of the measurement and buffer unit; (f) measurement principle of the measurement and buffer unit; (g) the relationship between the displacement of the moving plate and output voltage of the photo reflector.

The MAM comprises an adjustment unit and a measurement and buffer unit (Fig. 4). The adjustment unit is assembled in the fixed platform; the measurement and buffer unit is mounted on the mobile platform. Fig. 4 (b) and (c) show the internal structure and kinematic diagram of the adjustment unit. The gear train connecting the leadscrew to the stepper motor is installed in the adjustment rod. The adjustment rod has a ball swivel that forms a spherical joint when it is assembled in the fixed platform, and thus, it possesses 3 DOFs (i.e., rotations around the *x*-, *y*-, and *z*-directions). The connecting rod containing the lead nut is installed in the adjustment rod. The stepper motor rotates the leadscrew through the gear train, and the rotations of the leadscrew result in axial displacements of the connecting rod due to the meshing of the leadscrew and lead nut. Therefore, the axial movement of the connecting rod can be precisely controlled by the stepper motor.

Fig. 4 (d) and (e) show the internal structure and kinematic diagram of the measurement and buffer unit. The moving plate set in a bearing (BGS12-20, MISUMI, JP) can move in the axial direction and exert a force on the spring when the US probe is operated to perform scanning. The spring can be stretched or compressed when the moving plate moves up or down. A photo reflector (NJL5909RL-4, JRC, JP) mounted on the support plate measures the displacement of the moving plate. The measurement principle is shown in Fig. 4 (f) and (g).

The photo reflector is composed of a photo transistor and a LED. An aluminium evaporation sheet mounted on the moving plate is used to reflect the infrared rays emitted by the LED. The reflected rays will then be detected by the photo-transistor. The photo reflector outputs various voltages according to the collected rays that vary with distance changes between the photo reflector and aluminium evaporation sheet. Hence, the displacement of the moving plate can be obtained. Since the deformation of the spring has the same valve as the displacement of the moving plate, the force exerted on the moving plate (spring) can also be acquired by Hooke’s Law. The measurement and buffer unit has three functions: 1) measure the displacement of the moving plate that can be used as the input/feedback for pose adjustment of the US probe; 2) obtain the force applied on the moving plate that can be employed to calculate the operating forces and torques of the US probe; 3) cushion the undesired produced force using the deformation of the spring.

The adjustment unit and measurement and buffer unit are connected by a revolute joint and then work as a MAM (Fig. 4 (a)). Three parallel-connected MAMs can realize complex functions, including moving and rotating operations, torque measurement, and pose adjustment. Thus, the SAPM will be able to achieve compliant operations by maintaining operating forces and torques within safe ranges and automatically adjusting the pose of the US probe.

**Fig. 5:**
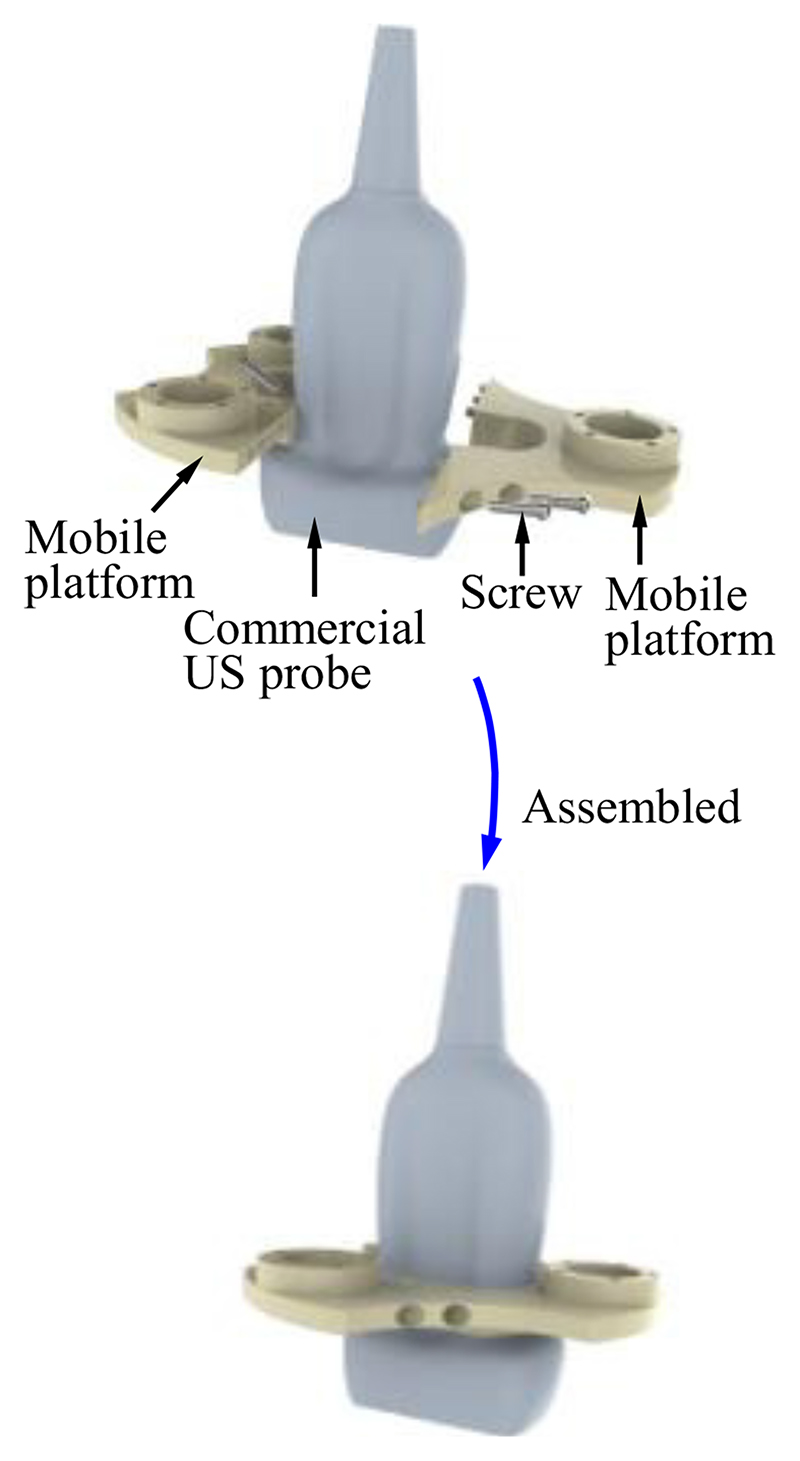
Mobile platform grasping the commercial ultrasound (US) probe.

The fixed platform is assembled in the robotic arm, and its connecting part needs to be combined with the end of the robotic arm (Fig.1 and 2). The structure of the connecting part varies with different types of robotic arms. In this research, the connecting part of the fixed platform is designed as a cylindrical sleeve that can be easily integrated with the robotic arm. To enable the mobile platform to grasp the US probe firmly (Fig. 2), the internal shape of the mobile platform is designed to be the same as the external shape of the US probe. The US probe is first scanned with a CT scanner and then its 3D mesh is extracted and imported into CAD software. A customized mobile platform can be developed based on this 3D model, and thus, the SAPM can apply to various US probes. In addition, to facilitate installation and disassembly of the US probe, the mobile platform is designed as a removable two-piece structure. As shown in Fig. 5, two pieces of the mobile platform can be assembled into one with screws.

#### C Control Principle and Workflow

As shown in Fig. 3, when the US probe rotates around the *x-* or *y-*directions, every measurement and buffer unit will be subjected to a force (*F*_*j*|*j*=1,2,3_) in its axial direction. The axial force increases when the measurement and buffer unit moves vertically toward the abdomen; it decreases when the measurement and buffer unit moves away from the abdomen. The axial forces *F_j_* can be used to evaluate the contact situation between the US probe and the abdomen, and therefore, we employ *F_j_* as criteria for the control of automatic pose adjustment. We preset forces *F*_*j*−0|*j*=1,2,3_ to define the ideal/predetermined contact situation between the US probe and the abdomen, and compare *F*_*j*|*j*=1,2,3_ to *F*_*j*−0|*j*=1,2,3_ to determine the movement change of each measurement and buffer unit. *F_j_* equal to *F*_*j*−0_ means measurement and buffer unit *j* has good contact with the abdomen. All three measurement and buffer units having good contact with the abdomen will indicate the US probe has a good pose for high-quality image acquisition. The pose change of the US probe can be determined by the movement of the three measurement and buffer units. Commonly, *F*_*j*−0|*j*=1,2,3_ could be set to one-third of the desired contact force for scanning to obtain ideal contact between the US probe and the scanned area. However, considering special operation habits or requirements for some sonographers, *F*_*j*−0|*j*=1,2,3_ could be preseted with different values or adjusted online to achieve various scanning poses.

**Fig. 6:**
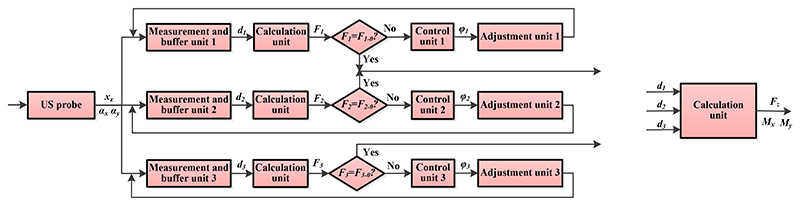
Workflow diagram of the SAPM. *x_z_*, *α_x_*, *α_y_* mean the displacement in the z-direction, and rotational angles around the *x*- and *y*-directions of the US probe, respectively; *d*_j_|*j*=1,2,3 are the moving distances measured by the measurement and buffer units *j*; *F*_*j*|*j*=1,2,3_ are forces applied to the measurement and buffer units *j*; *F*_*j*−0|*j*=1,2,3_ are predetermined forces for the measurement and buffer units *j*; *φ*_*j*|*j*=1,2,3_ are the rotatinal angles of the stepper motors *j* in the adjustment units *j*; *F_z_* indicates the force exerted on the US probe in the z-direction; *M_x_* and *M_y_* are the torques applied on the US probe around the *x*- and *y*-directions, respectively.

The workflow diagram is shown in Fig. 6. Since three MAMs are distributed in parallel, three similar processes (one for each MAM) are shown in the workflow. Changes in the abdominal contour result in various poses of the US probe. The pose change can be described by *x_z_*, *α_x_*, and *α_y_*, and be measured by the measurement and buffer units (i.e., *d*_*j*|*j*=1,2,3_). The forces (*F*_*j*|*j*=1,2,3_) exerted on the measurement and buffer units are then calculated and compared with the predetermined forces (*F*_*j*−0|*j*=1,2,3_). If the measured forces are equal to the predetermined forces, the MAM does not need to adjust the pose; if not, the adjustment unit will run based on the control signals (*φ*_*j*|*j*=1,2,3_) sent from the control unit. During the procedures, the force and torques exerted on the US probe (i.e., *F_z_*, *M_x_*, and *M_y_*) are obtained based on the displacement of the moving plate (*d*_*j*|*j*=1,2,3_). Determinations of *φ_j_*, *F_j_*, *F_z_*, *M_x_*, and *M_y_* are discussed and detailed in Section II-D.

#### D Mechanical Analysis and Kinematics Modelling

##### 1 Mechanical Analysis

When a force is exerted on the measurement and buffer unit, the spring will be compressed (Fig. 4 (d) and (e)). Based on Hooke’s Law, the forces applied to the measurement and buffer units can be obtained by

(1) $$F_{j}=k_{j}\delta_{j}$$

where *j* indicates the serial number of springs (*j*=1, 2, 3, and spring *j* is in measurement and buffer unit *j*), *k_j_* means the stiffness of spring *j*, and *δ_j_* is the deformation of spring *j*. The deformation of the spring has the same value as the moving plate, and thus, it can be measured by the photo reflector and written as:

(2) $$\delta_{j}=d_{j}$$

where *d_j_* is the displacement of the moving plate in measurement and buffer unit *j*.

**Fig. 7:**
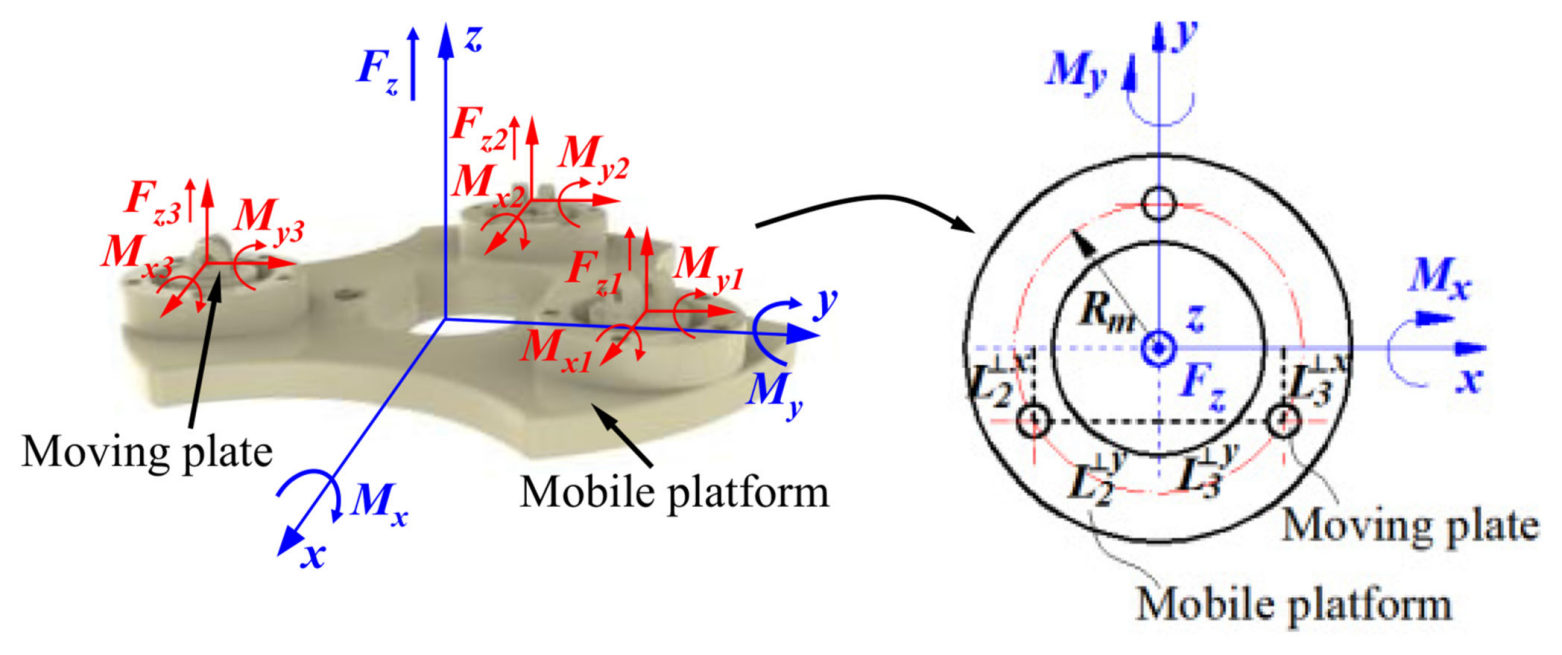
Diagram for force and torques analysis.

During the scanning, the US probe contacts the abdomen, and it is subjected to a force in the *z*-direction and torques around the *x*- or *y*-directions. As shown in Fig. 7, the forces and torques can be expressed as

(3) $$\left[\begin{array}{c} F_{z}\\ M_{x}\\ M_{y} \end{array}\right]=\left[\begin{array}{c} \sum F_{zj}\\ \sum M_{xj}\\ \sum M_{yj} \end{array}\right]$$

where *F_zj_* is the force exerted on measurement and buffer unit *j* in the *z*-direction, *M_xj_* means the torque exerted on measurement and buffer unit *j* around the *x-*direction, and *M_yj_* indicates the torque applied on measurement and buffer unit *j* around the *y-*direction. The forces and torques applied to the measurement and buffer units can be described as

(4) $$F_{zj}=k_{j}\delta_{j}$$

(5) $$M_{xj}=k_{j}\delta_{j}L_{j}^{⊥x}$$

(6) $$M_{yj}=k_{j}\delta_{j}L_{j}^{⊥y}$$

where $L_{j}^{⊥x}$ means the projection distance of measurement and buffer unit *j* on the *x*-axis and $L_{j}^{⊥y}$ is the projection distance of measurement and buffer unit *j* on the *y*-axis.

Substituting equations (2) and (4)-(6) into (3) results in

(7) $$\left[\begin{array}{c} F_{z}\\ M_{x}\\ M_{y} \end{array}\right]=M_{c}\left[\begin{array}{c} d_{1}\\ d_{2}\\ d_{3} \end{array}\right]$$

(8) $$M_{c}=\left[\begin{array}{ccc} k_{1} & k_{2} & k_{3}\\ -k_{1}R_{m} & 1/2k_{2}R_{m} & 1/2k_{3}R_{m}\\ 0 & -\sqrt{3}/2k_{2}R_{m} & \sqrt{3}/2k_{3}R_{m} \end{array}\right]$$

where ***M**_c_* is a calculation matrix for the force and torque measurement, and *R_m_* is the radius of the distribution circle of measurement and buffer units. Based on equations (7) and (8), the force and torques exerted on the US probe during the US scanning can be obtained.

##### 2 Kinematics Modelling

**Fig. 8:**
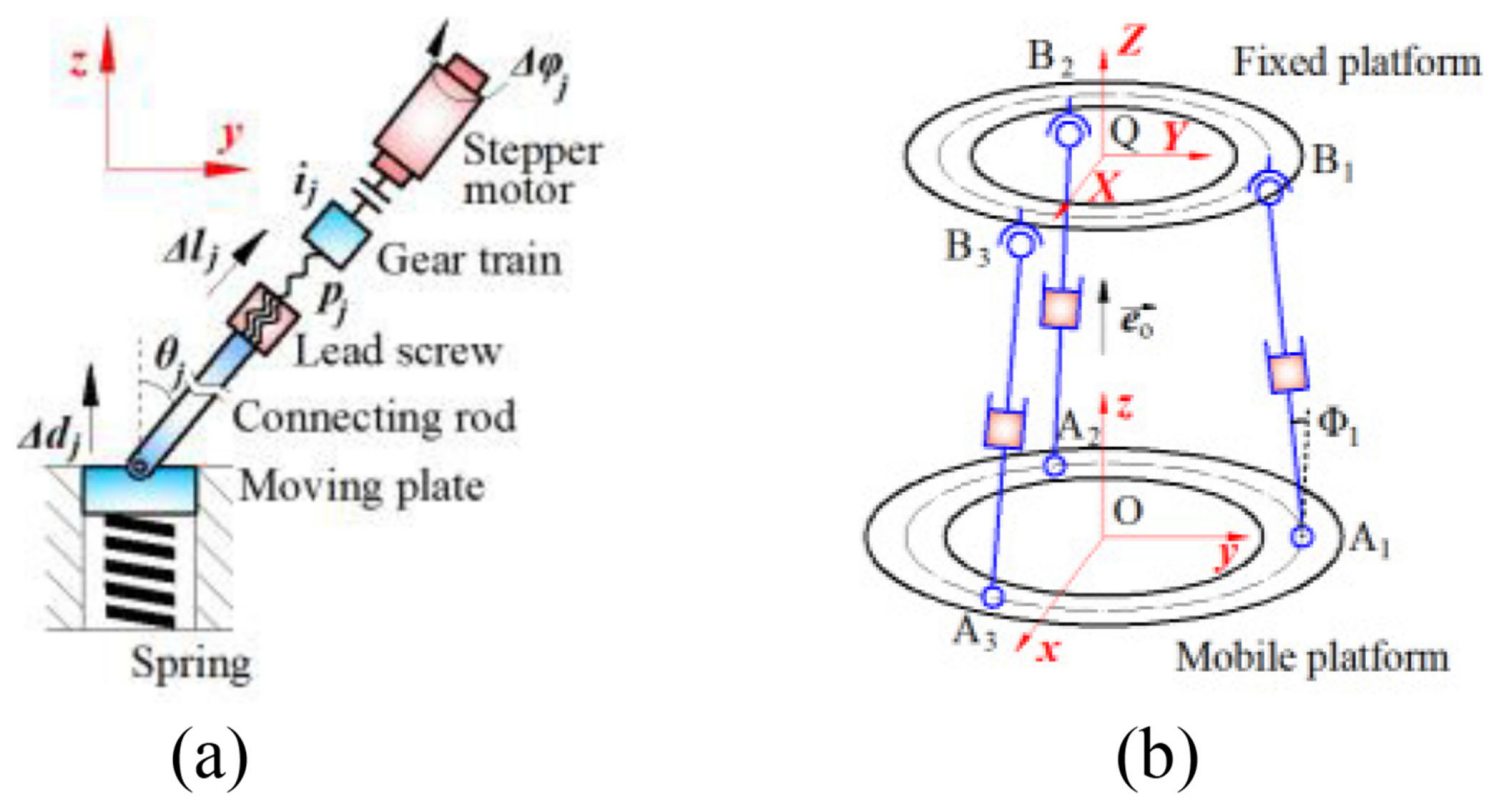
Kinematic diagrams of a single MAM (a) and parallel MAMs (b).

As discussed in Section II-C, we use *F*_*j*|*j*=1,2,3_ as criteria for the control of automatic pose adjustment. The forces *F_j_* can be obtained by equations (1) and (2), and they have linear relationships with the displacements of the moving plates *d_j_*. Thus, we investigate these relationships for the control of the adjustment units. As shown in Fig. 8 (a), the axial movement of the connecting rod results in the *z*-displacement of the moving plate, and the geometric relationship is

(9) $$\text{Δ}d_{j}=\text{Δ}l_{j}\text{cos}\theta_{j}$$

where *l_j_* is the axial movement of connecting rod *j*, and *θ_j_* represents the angle between the central axis of the moving plate and connecting rod *j*. Since the stepper motor rotates the leadscrew through the gear train, the axial movement of the connecting rod can be expressed as

(10) $$\text{Δ}l_{j}=\frac{p_{j}}{2\pi i_{j}}\text{Δ}\varphi_{j}$$

where *p_j_* means the lead of the lead screw *j*, *i_j_* indicates the transmission ratio of the gear train *j*, *φ_j_* is the rotational angle of the stepper motor *j*. Substituting equation (9) into equation (10) results in

(11) $$\text{Δ}\varphi_{j}=\frac{2\pi i_{j}}{p_{j}\text{cos}\theta_{j}}\text{Δ}d_{j}.$$

In addition, as shown in Fig. 8 (b), based on the space vector calculation, there is

(12) $$\vec{L_{A_{j}B_{j}}}\cdot \vec{e_{O}}=\left|\vec{L_{A_{j}B_{j}}}\right|\left|\vec{e_{O}}\right|\text{cos}\;{\text{Φ}}_{j}$$

where $L_{A_{j}B_{j}}$ means a vector formed by point *A_j_* and the connection point of adjustment rod *j* and the fixed platform (i.e., point *B_j_*), $\vec{e_{O}}$ is a normal vector of the mobile platform, and Φ*_j_* is the angle between $L_{A_{j}B_{j}}$ and $e_{O}$. According to the geometric relationship, there is

(13) $${\text{Φ}}_{j}=\theta_{j}.$$

Substituting equations (12) and (13) into equation (11) results in

(14) $$\text{Δ}\varphi_{j}=K\text{Δ}d_{j}$$

(15) $$K=\frac{2\pi i_{j}\left|\vec{L_{A_{j}B_{j}}}\right|\left|\vec{e_{O}}\right|}{p_{j}\vec{L_{A_{j}B_{j}}}\cdot \vec{e_{O}}}$$

where *K* is the transformation coefficient for the rotational angle and displacement. Hence rotational angles for the stepper motor control can be obtained by equations (14) and (15).

##### E Prototjpe and Further Application Considerations

**Fig. 9:**
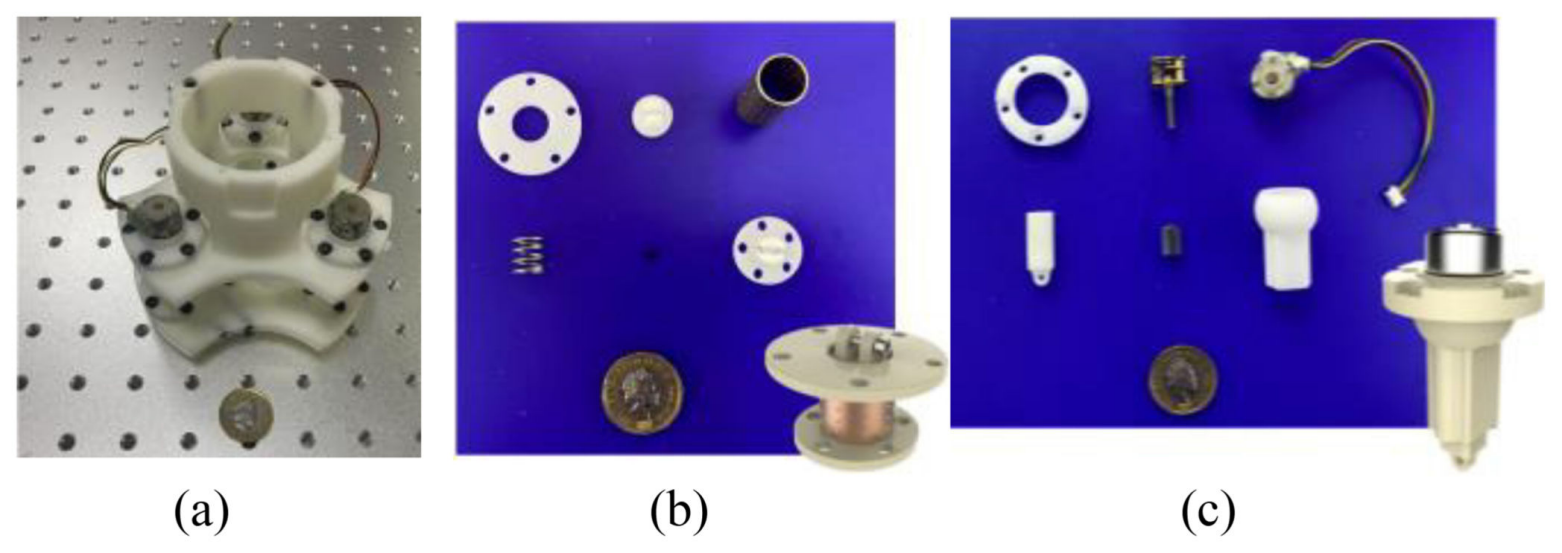
Prototype of the proposed SAPM: (a) assembled SAPM; (b) components of the measurement and buffer unit; (c) components of the adjustment unit.

Fig. 9 shows the prototype of this proposed SAPM, including the assembled SAPM and crucial components. The prototype was fabricated through 3D printing. The SAPM has dimensions of 114 mm × 114 mm ×148 mm. The overall size of the SAPM is determined by the dimensions of the US probe and the clinical requirements, and it will have a great volume when a big workspace is required. The SAPM weighs 192 g and thus it can be easily integrated into the robotic arm.

Due to the simple structure, low cost, and easy control, the proposed SAPM has potential use in some other engineering or medical applications that need force and torque measurement or pose adjustment, especially for operations involving safety or requiring high concentration. In addition, the proposed SAPM can provide compliant operations similar to soft robots that have soft contact with the objects and can buffer unexpected operating forces (discussed in Section III-E). The workspace of the SAPM can be increased by using longer adjustment rods and lead screws, and the load capacity and positioning accuracy will be improved by employing motors with high accuracy and great power and gear trains with high reduction ratios. With these substitutions, the SAPM could be applied to various scenarios.

## III Performance Evaluation

### A Motion Acquisition

**Fig. 10:**
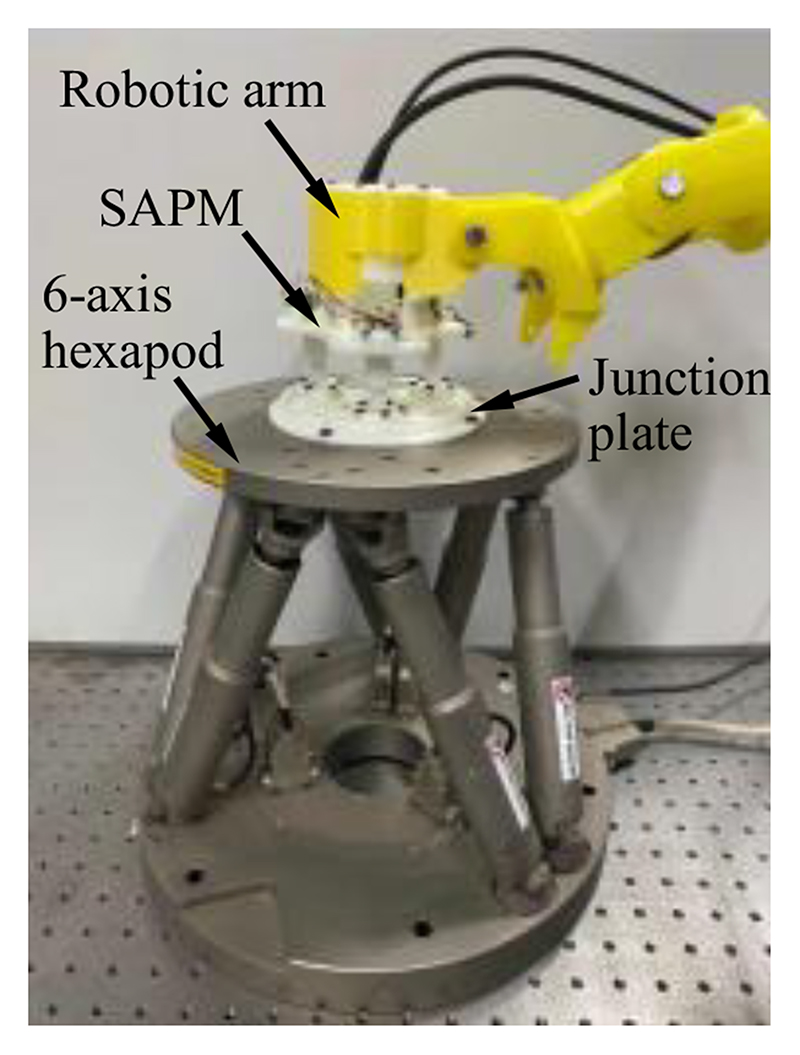
Experimental setup for motion acquisition experiments.

The SAPM has three measurement and buffer units capable of displacement measurement. In this section, accuracy and precision evaluation experiments were conducted to evaluate the displacement measurement performance of the three measurement and buffer units. Fig. 10 shows the experimental setup. A 6-axis hexapod (H-840, Physik Instrumente (PI), GER) was employed to generate the desired displacements, and a hexapod motion controller (C-887.52, PI, GER) was used to control the 6-axis hexapod. The SAPM mounted on a robotic arm was connected to the 6-axis hexapod through a junction plate, and thus, the mobile platform of the SAPM will have the same displacements and rotations as the upper plate of the 6-axis hexapod. In the accuracy evaluation experiments, the 6-axis hexapod produced movements with 0.4-mm increments from 0 mm over a range of 3.2 mm in the *z*-direction. All three measurement and buffer units measured the positional data at each increment and these operations were repeated nine times. In the precision evaluation experiments, the 6-axis hexapod moved to the 1.6-mm position in the *z*-direction nine times, and displacements were also recorded by the measurement and buffer units. Corresponding standard deviations of the error were calculated.

The accuracy and precision of the three measurement and buffer units are 0.04±0.11 mm, 0.04±0.10mm, and 0.04±0.12 mm, respectively. The performance evaluation shows the ability of the measurement and buffer units to measure the displacement of the moving plate (US probe), which will provide a basis for mechanical measurement and automatic pose adjustment. The performance of the mechanical measurement and automatic pose adjustment were evaluated in Sections III-C and III-D.

### B Workspace

**Fig. 11:**
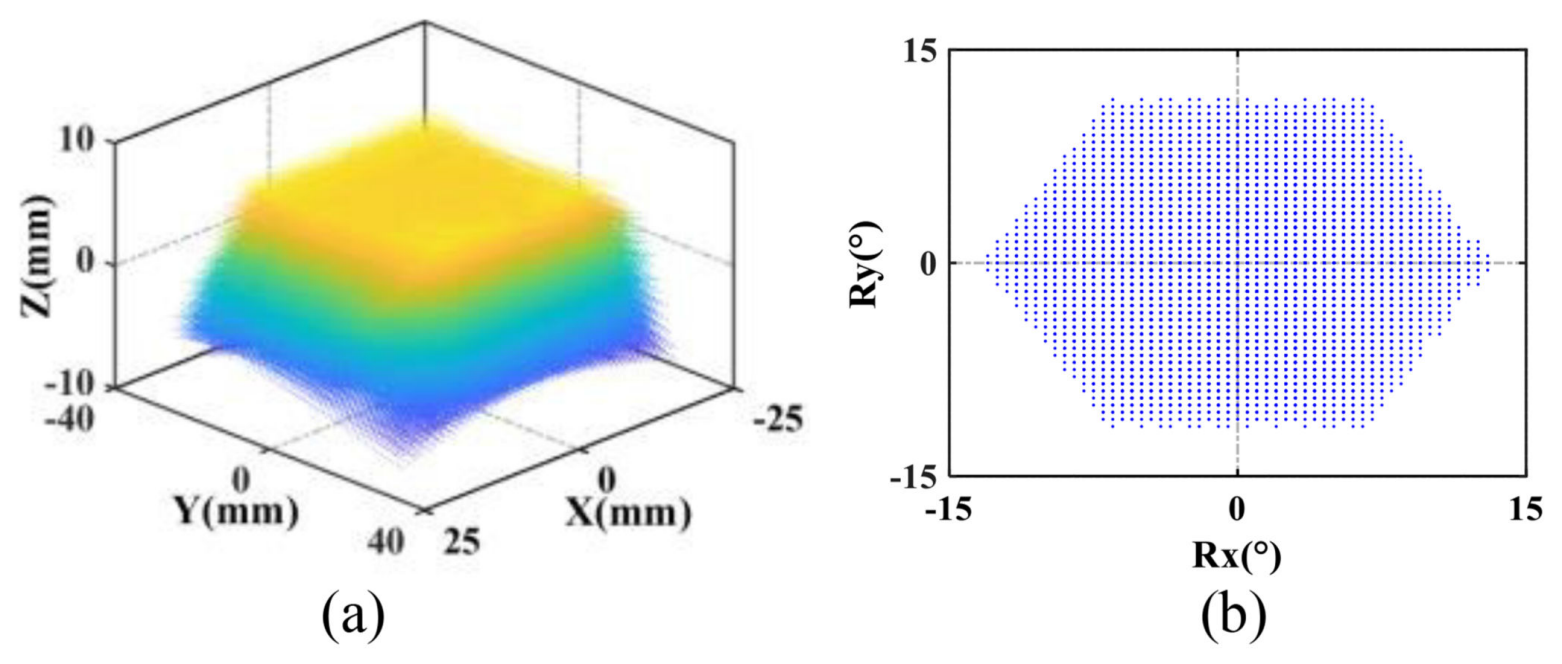
Attainable space (a) and rotation range (b) of the US probe.

In order to verify whether the attainable motions of the SAPM satisfy the imposed clinical requirements for the US probe motion, the workspace of the SAPM was investigated. As shown in Fig. 4 and 8 (b), three MAMs are connected in parallel, and they have three revolute joints, prismatic joints, and spherical joints. We regarded the bottom surface of the US probe that can emit ultrasound as the target and calculated the attainable spatial positions and rotational angles by using MATLAB. The bottom surface of the US probe has dimensions of 30mm×50mm and is 45mm away from the mobile platform of the SAPM. As shown in Fig. 8 (b), the distribution circle diameter of the three parallel MAMs (i.e., the circle formed by points *A*_*j*|*j*=1,2,3_ and by points *B*_*j*|*j*=1,2,3_) is 83 mm. The variation range of rod *A*_*j*_*B*_*j*|*j*=1,2,3_ length is 30.2mm-44.8mm. The maximum rotation angles of the spherical joints and the revolute joints are 80° and 70°, respectively. Fig. 11 shows the workspace of the proposed SAPM. The dots in Fig. 11(a) were marked in various colours to increase the visualization of the attainable space (the colour changes with the Z value). The SAPM can move within 14.6mm in the *z*-direction, and rotate within ±13° and ±11.5° around the *x* and *y*-directions, respectively. Thus, the proposed SAPM satisfies the clinical requirements presented in Section II-A.

### C Force and Torque Measurement

**Fig. 12:**
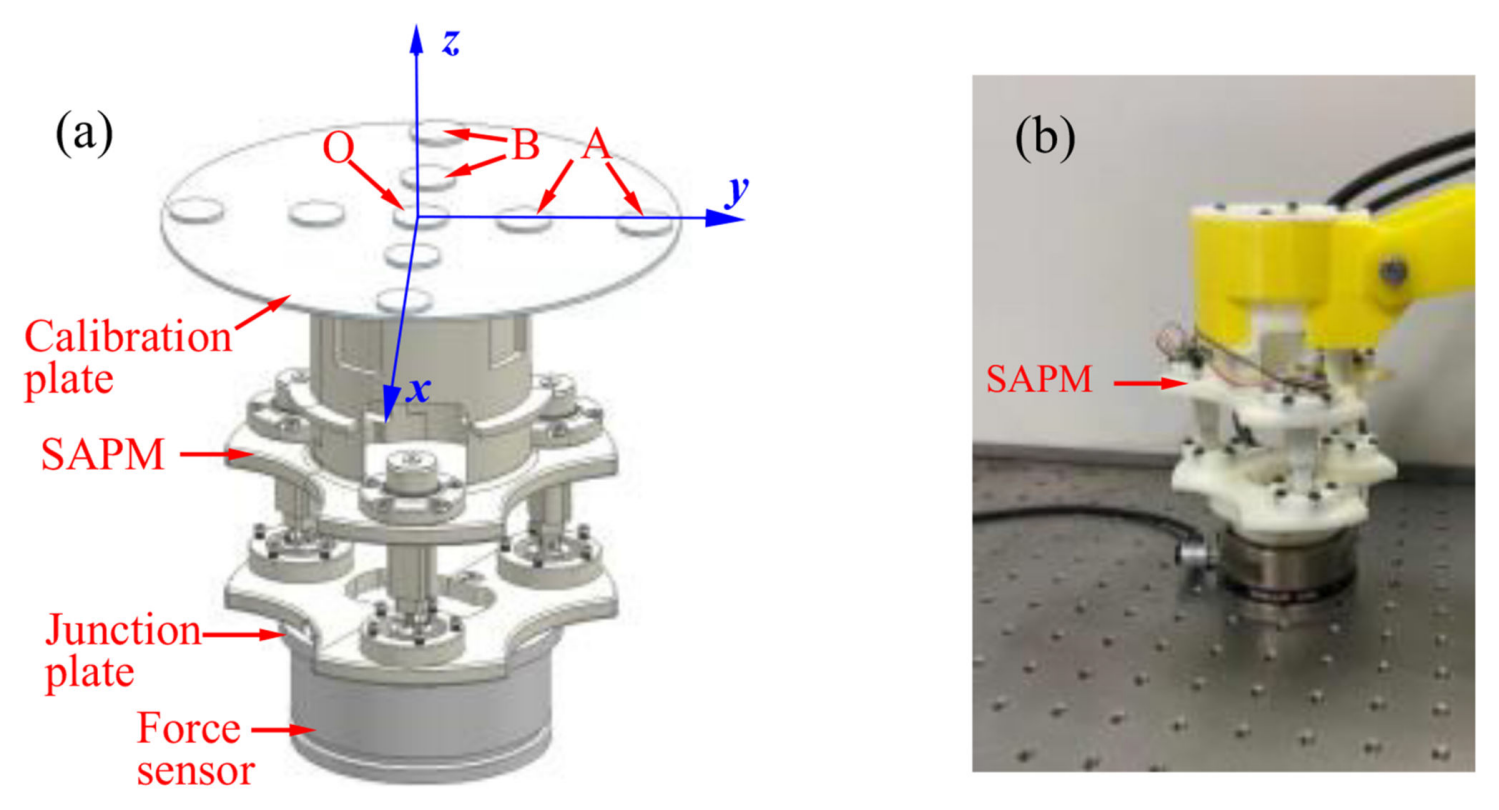
Experimental setup for calibration experiments (a) and performance evaluation experiments (b).

To evaluate the measurement performance of the SAPM, we first conducted the calibration experiments to determine the calibration parameters, and then exerted random forces and torques on the SAPM and observed the mechanical response. The experimental setup is shown in Fig. 12. A 6-axis force sensor (Gamma, ATI Industrial Automation, Inc., USA) connected to the SAPM through a junction plate was used to measure the forces and torques applied to the SAPM. In the calibration experiments, a calibration methodology proposed in our previous research [27] was employed in this experiment. This methodology obtained the input and output of the SAPM by three loading modes, and the exerted forces and torques in the three modes are *F_z_*, *F_z_* and *M_x_*, and *F_z_* and *M_y_*, respectively.

Weights are placed in different places to produce various forces and torques. As shown in Fig. 12 (a), *F_z_* was generated when weights were put at position O; *F_z_* and *M_x_* were produced when weights were located at position A; *F_z_* and *M_y_* were generated when weights were placed at position B. During each loading procedure, output voltages of three photo reflectors were recorded, and corresponding ground-truth forces and torques were acquired by the 6-axis force sensor. Based on the recorded data, a calibration matrix can be obtained by using the Least Squares Regression method [28]. After the calibration, in the verification experiment, we operated the SAPM randomly within the force and torque measurement range. The applied forces and torques were acquired by both the SAPM and the 6-axis force sensor. The measured values were compensated since there were various moment arms for the SAPM and 6-axis force sensor when forces were applied to the calibration plate.

According to the experimental data, the calibration matrix was obtained, and it is

(16) $$M_{M}=\left[\begin{array}{ccc} 3.990 & 3.816 & 3.808\\ -171.579 & 76.323 & 79.968\\ 2.432 & -132.178 & 138.502 \end{array}\right].$$

The matrix condition number can characterize the numerical stability and it can be calculated by

(17) $$\chi_{M}=\left\|M_{M}\right\|\cdot \left\|M_{M}^{-1}\right\|.$$

Normally, the greater the condition number, the more singular the matrix is, and the matrix becomes ill-conditioned when the condition number is larger than 1000. The ill-conditioned matrix is very sensitive to input, and with the ill-conditioned matrix, small noise will lead to drastic changes in measured force and torque. Based on equations (16) and (17), the condition number of the calibration matrix equals 30.53, which means this calibration is well-conditioned. Therefore, the SAPM can measure the forces and torque by

(18) $$\left[\begin{array}{c} F_{z}\\ M_{x}\\ M_{y} \end{array}\right]-M_{M}\left[\begin{array}{c} V_{1}\\ V_{2}\\ V_{3} \end{array}\right]$$

where *V*_1_, *V*_2_, and *V*_3_ mean the output voltages of the three photo reflectors.

**Fig. 13:**
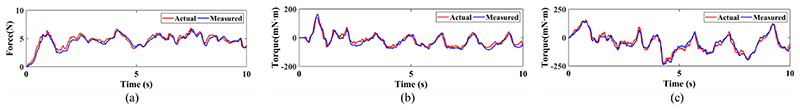
Force and torque measurement: (a) forces in the *z*-direction; (b) torques around the *x*-direction; (c) torques around the *y*-direction.

More specifications of the proposed SAPM are shown in TABLE I. The measurable ranges of the SAPM are larger than the design requirements, i.e., the axial force of 8.01 N and torques of 220 mN·m around the *x*- and *y*-directions, and thus, the proposed SAPM satisfies the clinical requirements. Although the linearity, repeatability, and hysteresis of the proposed SAPM cannot be obtained by this calibration method, this method provides a straightforward calibration process and takes a short time. Fig. 13 shows the results of the verification experiments. The red lines represent the forces or torques measured by the ATI force sensor; the blue lines indicate the forces or torques acquired by the SAPM. The maximum errors for the three directions are 1.04N, 44.21mN·m, and 50.38mN·m, respectively. These experiments show the ability of the SAPM to measure the forces and torques of the US probe that can be used as operation status monitoring and early warning during the scanning.

### D Automatic Adjustment

The proposed SAPM is able to automatically adjust its pose to maintain contact forces and torques within a safe range and obtain a suitable pose for high-quality image acquisition. To evaluate the automatic adjustment performance of the SAPM, we conducted experiments to observe its response and force/torque changes when the scanning contour varied. The experimental setup was the same as Section III-B (Fig. 12 (b)). The ATI force sensor was operated randomly to simulate the possible changes in the scanning contour. The contact force was set to 6N to simulate the safe operation, and the contact toques were set to 0 mN·m to guarantee the US probe was in good contact with the scanned area (Fig. 3). Contact forces, contact torques, and rotational angles of the stepper motors in the SAPM (Fig. 4(a)) were recorded for observation.

**Fig. 14:**
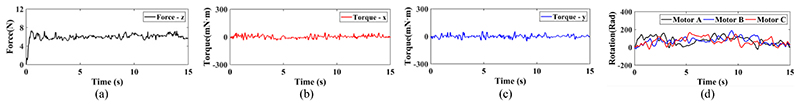
Results in the automatic adjustment experiments: (a) forces in the *z*-direction; (b) torques around the *x*-direction; (c) torques around the *j*-direction; (d) rotations of the stepper motors.

The simulated scanning contour changed during the procedure. The SAPM captured these changes and adjusted its pose (US probe pose) to adapt to the contour of the scanned areas through the rotations of stepper motors (Fig. 14 (d)). As a result, the contact forces and torques were maintained at approximately constant values (Fig. 14 (a)-(c)). The constant contact forces show the operation was performed within safe thresholds; the constant torques indicate the US probe had good contact with the scanned area; the force and torque fluctuations mean the US probe was in the adjustment process. The constant contact forces and torques are conducive to obtaining high-quality US images and will bring comfort to patients. Since the SAPM can automatically adjust its pose and maintain constant contact forces and torques within a safe range, sonographers do not need to pay attention to the value of the operating forces/torques and the contours of scanned areas, thus reducing the workload of sonographers and improving operation safety and efficiency.

In Fig. 14 (a)-(c), the maximum force error was 1.32N; the maximum torque errors around the *x*- and *y*-directions were 47.56mN·m and 55.96mN·m, respectively. The contact forces and torques were not strictly constant and changed within a certain range. However, these operating forces and torques limited within a range can ensure scanning safety. On the other hand, using high-speed motors can increase the contact force and torque generation accuracy, and the error variation range of operating forces and torques will be reduced.

### E Phantom Experiment

**Fig. 15:**
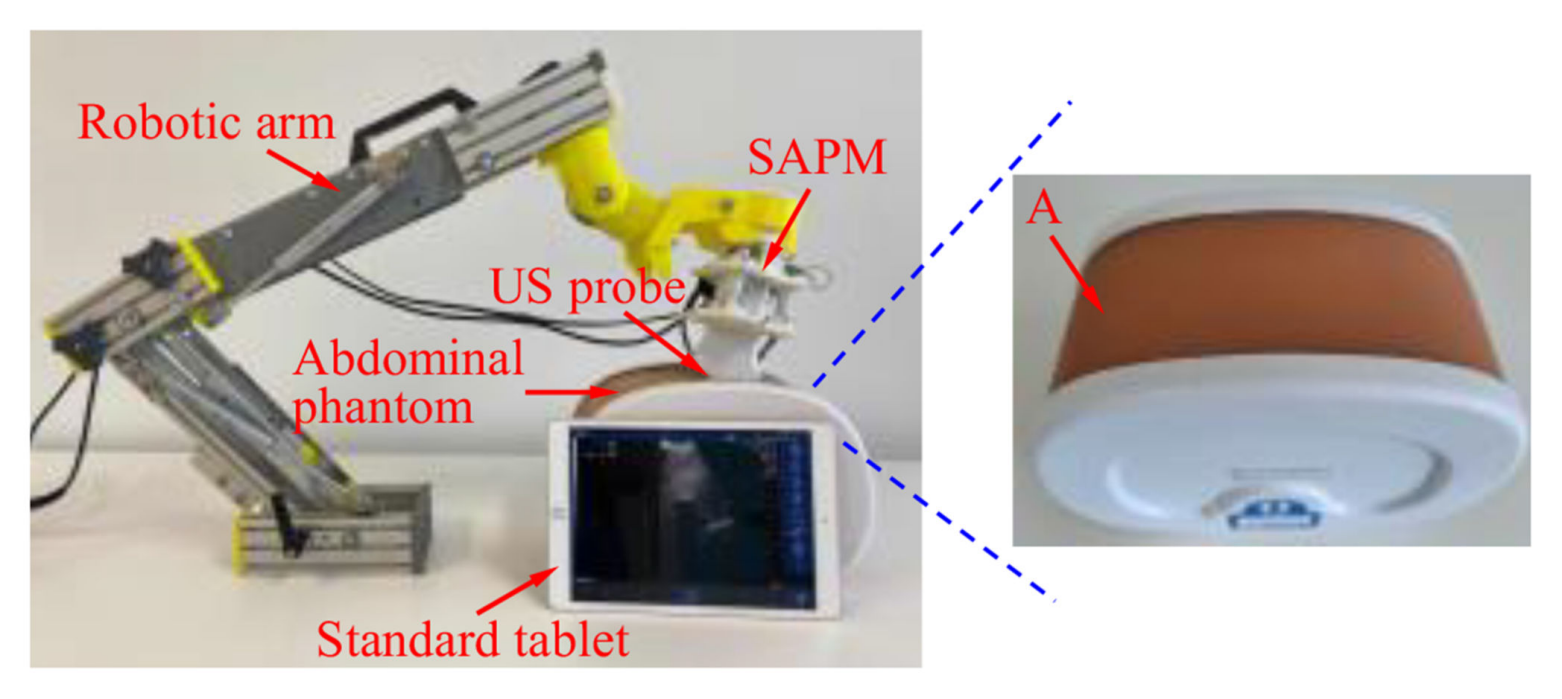
Setup for the phantom experiment.

To test whether the proposed SAPM can automatically adjust a US probe pose to obtain US images, phantom experiments were carried out, and the experimental setup is shown in Fig. 15. A US probe (CProbe, Sonostar Technologies Co., Ltd., CN) was installed in the SAPM mounted on a robotic arm. An abdominal US phantom (057A, Computerized Imaging Reference Systems, Inc., USA) simulated the scanned human abdomen. The US probe was placed where the contour changes (i.e., position A in Fig. 15 (b)). The SAPM started to work as the procedure in Fig. 3. US images were acquired by the US probe when the SAPM completed the pose adjustment and then transmitted to a standard tablet (iPad) and displayed on its screen. In order to have a comparison, the same procedure was performed manually.

**Fig. 16:**
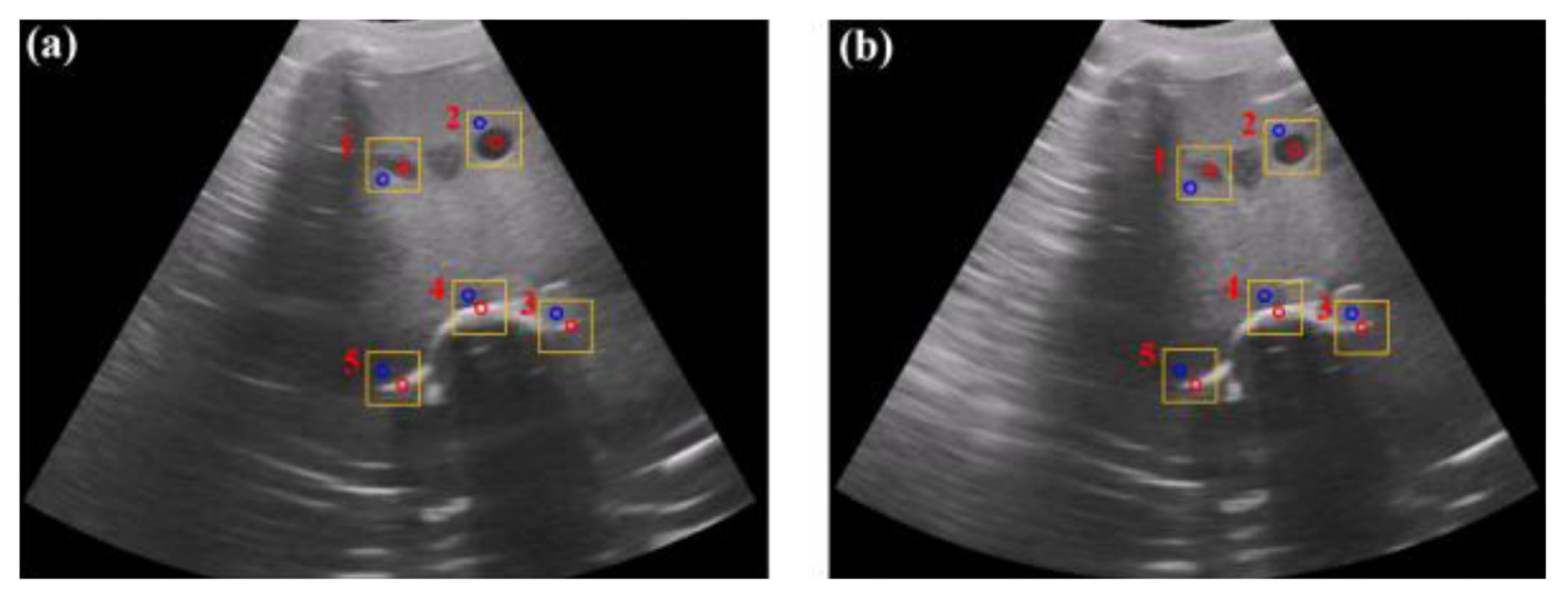
US images acquired by the SAPM (a) and the manual operation (b). Five regions of interest (ROIs) (in the red circle) and five contrast regions (in the blue circle) were selected for local contrast-to-noise ratio (LCNR) calculation.

**Fig. 17:**
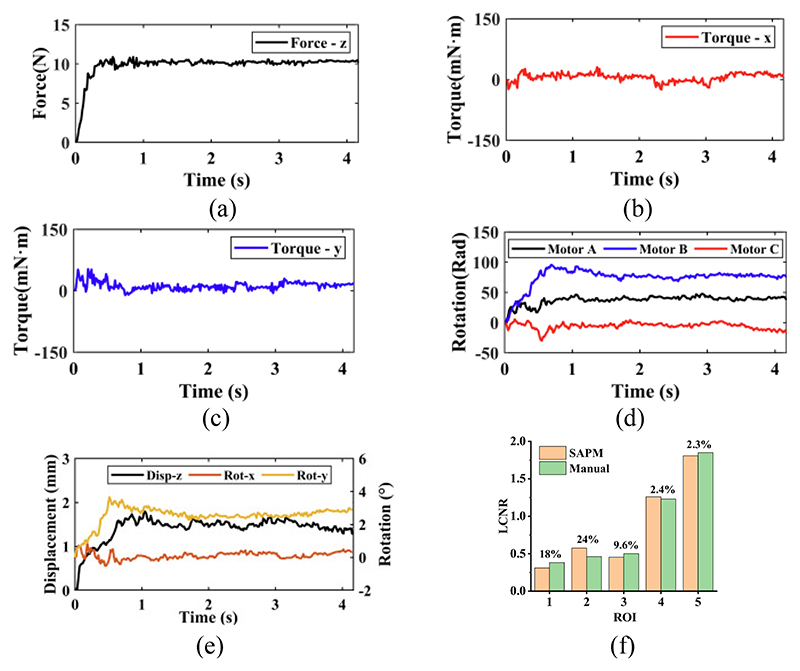
Experimental data in the phatom experiment: (a)-(c) operating froces and torques; (d) rotations of stepper motors; (e) displacements and rotations of the US probe; (f) comparison of the local contrast-to-noise ratio (LCNR) for selected ROIs in the SAPM- and manually-acquired images.

The US images acquired by the SAPM and manual operation are shown in Fig. 16. Fig. 17 (a)-(e) show the force and motion data in the phantom experiment. Since the US probe pose cannot be obtained directly without external sensors, the rotations of the stepper motors recorded by their own encoders were employed to calculate the pose change during the experiment (Fig. 17(e)). In Fig. 17, before about 0.8s, the US probe started to contact the scanned area and changed its poses to obtain good contact with the scanned area. During this process, the operating forces and torques around the *y*-direction varied rapidly, and the poses of the US probe changed accordingly with the rotations of the stepper motors. The maximum force and torque errors occurred during the adjustment process: the maximum force error was 0.88N; the maximum torque errors around the *x*- and *y*-directions were 30.41mN·m and 52.83mN·m, respectively. After the adjustment, the US probe was in good contact with the abdominal surface, and all the motion changes became smooth, including the rotations of the stepper motors and the poses of the US probe. Accordingly, the operating forces and torques varied slightly.

In Fig. 16, the spine, aorta, and lesion structures of this abdominal US phantom are clearly visible both in the SAPM- and manually-obtained images. To quantify the similarity of the acquired images, we employ a local contrast-to-noise ratio (LCNR) by

(19) $$LCNR=\frac{\left|I_{R}-I_{b}\right|}{I_{b}}$$

where *I_R_* indicates the mean intensity of the region of interest (ROI) and *I_b_* represents the mean intensity of the background for ROI. We selected five ROIs (in the red circle) from both SAPM- and manually-obtained images in Fig. 16, and five contrast regions (in the blue circle) as the background region. The comparison of the LCNR is shown in Fig. 17(f). We found that these five ROIs have similar LCNRs: some of the ROIs in the SAMP-obtained image have higher LCNRs than those in the manually-obtained image, while the others have lower LCNRs; the maximum and minimum relative differences are 24% and 2.3%, respectively. This experiment shows that the SAPM could have a similar operation performance as the manual operation in guiding US probes for imaging acquisition.

During actual scanning, undesired motions would be produced by patients, robotic arm, and other possible contingencies and are exerted on the US probe. Undesired forces generated by these motions will bring discomfort and potential injury to patients [6]. The stiffness of the visceral contents (*k_v_*) can be obtained by

(20) $$k_{v}=\frac{ES}{h}$$

where *E* is Young’s modulus for visceral contents, *S* means contact area, and *h* is the tissue thickness. The stiffness of the proposed SAPM (*k_s_*) is determined by

(21) $$k_{s}=\sum k_{j}$$

where *k_j_* is the stiffness of spring *j* in the MAMs. When the SAPM is used, the combined stiffness of the whole system (SAPM and visceral contents) can be determined by

(22) $$k_{c}=\frac{k_{v}k_{s}}{k_{v}+k_{s}}.$$

Since *k_v_* > 0 and *k_s_* > 0, there will be *k_c_* < *k_s_* and *k_c_* < *k_v_*. The combined stiffness of the whole system is smaller than that of both the SAPM and visceral contents. When undesired motions (△*s*) are applied, forces will be produced by *F* = *k*△*s*. This means lower stiffness results in smaller produced forces.

Assuming a circular contact of 10 mm radius with a tissue thickness of 50 mm, the stiffness of the visceral contents is acquired as *k_v_*=52.88 N/mm, according to equation (20) and approximate Young’s modulus of 8.42 kPa for visceral contents [29]. The stiffness of the spring in the MAMs is 2.9N/mm, and based on equations (21) and (22), we can obtain *k_s_*=8.7N/mm and *k_c_* =7.47 N/mm. Assuming the displacement of the undesired motions is 3mm (Δ*s*=3mm), the undesired produced force without using the SAPM is 158.64 N, while the undesired produced force using the SAPM is only 22.41N. Therefore, the proposed SAPM can cushion the undesired produced forces like soft robotics. Considering the reduction in contact force when exposed to an involuntary patient or clinician motion, it can be assumed that using the proposed SAPM instead of rigid end-effectors could significantly improve operation safety.

## IV Conclusion

In this paper, a novel SAPM was developed and its performance was evaluated through experiments. This proposed SAPM can automatically adjust the US probe pose to adapt to various contours of scanned areas, provide approximate constant operating forces and torques, measure operating forces and torques, and cushion undesired produced forces. This SAPM has simple structures, low cost, and good expandability. This proposed solution can be integrated with current ultrasound robots or commercially-available robotic arms, such as [19], [24]–[25], and potentially be extended to some other engineering or medical applications. Experimental results demonstrated it could satisfy the clinical requirements of fetal imaging and have the potential for use. In our future work, we will further explore its application features through volunteer experiments. On the other hand, since compliant mechanisms have been proven to have many outstanding advantages compared with rigid-body mechanisms [30]–[31], we will also try to introduce the soft-body counterpart to our research and investigate its potential application.

## Figures and Tables

**Table 1:** Accuracy of The Proposed Sapm

Type	Measurable ranges	Maximum error	Root-mean-square error
*F_z_*	-11N-+16 N	29.4%	0.79 N
*M_x_*	±350 mN·m	34.2%	38.25 mN·m
*M_y_*	±300 mN·m	35.6%	40.11 mN·m
